# Reevaluating the role of amyloid *β*-peptides in Alzheimer’s disease: from pathogenic agents to protective chelation mechanisms

**DOI:** 10.3389/fneur.2025.1550709

**Published:** 2025-03-28

**Authors:** Franco Cavaleri

**Affiliations:** Biologic Pharmamedical Research, Vancouver, BC, Canada

**Keywords:** Alzheimer’s disease, amyloid *β*-peptides, BACE1, heavy metal toxicity, oxidative stress, neuroprotection, tau hyperphosphorylation, neurodegeneration

## Abstract

Alzheimer’s disease (AD) is a multifaceted neurodegenerative disorder with complex etiology, often associated with histological markers of oxidative stress, inflammation, and disturbances in calcium homeostasis. Traditionally, amyloid *β*-peptides (Aβ) have been considered key contributors to these pathological processes. However, emerging evidence suggests a protective role for Aβ and the enzymes involved in its production. This article further explores the hypothesis published by us a decade before that posits amyloid *β*-peptides and the β-secretase enzyme (BACE1) are part of an intentionally designed cellular defense mechanism against metal toxicity. This challenges the conventional understanding of their roles in AD pathogenesis. It is not until this BACE1 system, primarily the associated amyloid plaque deposit sites, are saturated with heavy and other metals and the exposure to these cations continues to influx oxidative ions into the brain, do the indications of neurodegeneration begin to become symptomatic. Until this metal oversaturation takes place, the system – Aβ and the enzymes involved in its production and conveyance – keeps the oxidative potential of the metal toxins sequestered extracellularly and out of the way of the neuron’s intracellular activities.

## Introduction

Alzheimer’s disease (AD) is characterized by progressive neurodegeneration and cognitive decline, with a significant increase in prevalence expected in the coming decades. Current models primarily implicate amyloid *β*-peptides (Aβ) in the disease’s progression, associating them with various cellular dysfunctions such as oxidative stress, inflammation, and calcium dysregulation (1, 2). The enzyme *β*-secretase (BACE1), responsible for cleaving amyloid precursor protein (APP), has been viewed as a pathological driver in this context (3–5). However, this article expands on our previously published papers (6, 7) proposing a novel perspective; positioning Aβ and BACE1 as protective countermeasures against heavy and other metal toxicity. In this brief article we expand on the original proposal to dive deeper into the mechanism tied to the amino acid sequence of the Aβ peptide; a sequence that varies and whose functionality varies based on the different cleavage points by different enzymes. We attempt to show here how these different amino acid sequences relate to metal chelation and hydrophobic moieties that facilitate intercellular amyloid plaque formation. In the proposed paradigm, this biological activity to sequester the dangers of the highly oxidative metals is an intended design.

The brain represents approximately 2 % of the body’s weight but uses as much as 20% of the body’s oxygen consumption (8). In addition, the density of the polyunsaturated fat and cholesterol mass in the membranes of the 100 trillion brain cells and the associated myelin is intensely more than that which we find in the cells of the rest of the body (7). The brain can house as much as 25% of the body’s cholesterol (8), yet again it represents only 2 % of the body’s mass. These concentrated substrates (and structural components) can be highly vulnerable to oxidation as they neutralize free radical stress (9) in the brain where oxygen consumption is at such high rates. The polyunsaturated fatty acid-rich environment of the brain, mainly arachidonic acid and docosahexaenoic acid is critical to more than just membrane structure. They are critical components of signaling mechanisms, synaptic function, neurogenesis and more (10). These polyunsaturated fats have a profound influence on brain function and are implicated in mood disorders, depression, bipolar, schizophrenia, attention deficit hyperactivity disorder and also AD (11). Research also irrefutably demonstrates that oxidation of these fats or other alteration of their chemistry results in altered signaling that circles back to implications related to all these behaviors as well as structure and function disorders, including AD (10).

**Figure 1:**
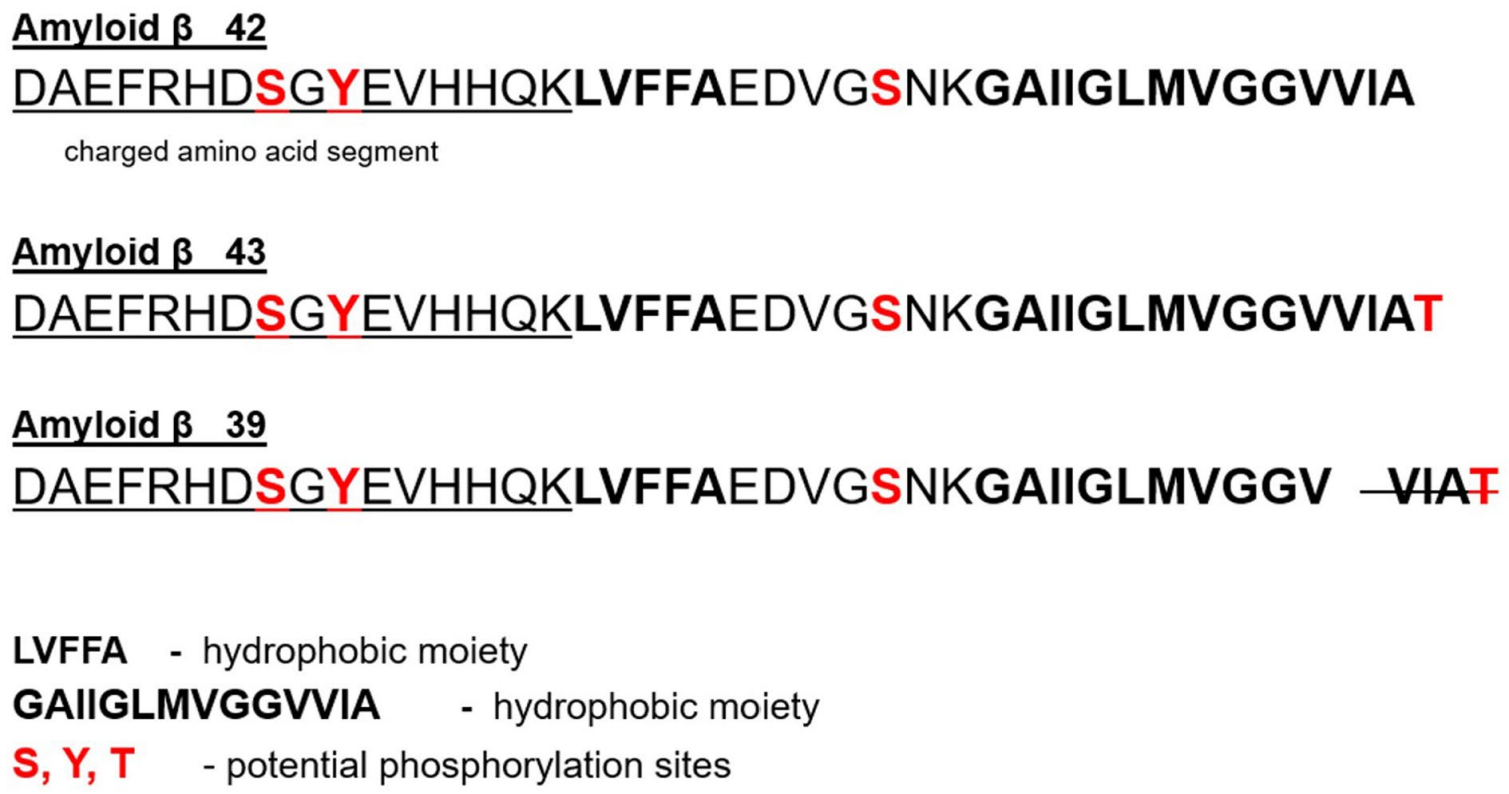
Showcases the different domains of the BACE1-Generated (*β*-Secretase Enzyme) A*β* peptide associated with metal ion chelation and those with hydrophobic activity known to facilitate aggregation and Beta sheet formation (46). In particular, the side-by-side histidine (13 and14) residues formed bridges through metal cross-linking providing insight on another feature promoting the aggregation of A*β* protein referred to in the medical community as ‘pathogenic aggregation’ (50), but considered by this paradigm to be an intended biological design to protect the brain from oxidation (including Fenton Reaction).

As such, with this density of ‘volatile fuels’ that are vulnerable to oxidation in an environment that is exposed to an extraordinary oxygen burden, we would expect extraordinary precautions are naturally inbuilt to protect the tissues and interactions from this vulnerability. BACE1 activity is directly related to escalated inflammatory activity – NF-kB signaling (12–14). This may be a designed protective response to inflammation exacerbated by oxidation which itself is facilitated by the Fenton reaction or oxidative activity facilitated by other cations. Oxidation promotes inflammation and vice versa (15, 16). The BACE1 activity may not at all be nefariously pathological. It is more likely protective as it generates the unique Aβ peptide described in detail in Figure 1 with a greater capacity to chelate, capture and sequester the danger that these metals represent to this highly volatile environment in the brain. It should be expected that the highly vulnerable conditions of the structural and signaling components in the brain are at incremental risk due to the enhanced metabolic activity of the neuron and the dependence on higher oxygen consumption than anywhere else in the body.

While the BACE1 system can provide compensatory protection, persistent exposure to these metals eventually overwhelms the successful countermeasure and in due time, collapse of the protective mechanism results in disease. Heavy metals seem to play a pivotal role in this context. Upon analysis of senile plaques, Sakae et al. observe the presence of aluminum in the plaques while no aluminum was found in the extracellular space or in the cytoplasm of the studied nerve cells (17). A review of multiple studies into aluminum’s association with neurodegeneration relating to AD demonstrates aluminum is noted to be associated with the development of AD (18). Aluminum is shown to form structured aggregates with Aβ and results in high neurotoxicity (19). Lead exposure results in increased Aβ fibril formation and plaque deposition (20). Other work also shows exposure to lead results in amyloidogenic activity and AD-associated pathology (19). AD research has also shown a possible association between mercury and AD (21) where mercury exposure could increase the risk of developing AD (22). Cadmium interacts directly with Aβ forming aggregates and is considered a possible risk factor in AD as well (23). Heavy and other metals are central to the AD pathology.

### Background and rationale

Despite substantial research, the precise mechanisms underlying AD remain elusive. Histological analysis of AD-affected brains reveals several cellular dysfunctions, many linked to Aβ. While the prevailing hypothesis suggests that Aβ contributes to neurodegeneration, recent findings indicate that Aβ might play a protective role supporting our decade long proposal. This current article builds on our previous work (6) proposing that the upregulation of BACE1 and subsequent production of A*β* are part of a cellular response to inflammation initiated by the oxidative heavy or other metal toxicity, a significant but often overlooked factor in sporadic AD as well (24).

## The hypothesis

Amyloid β-peptides and the β-secretase enzyme (BACE1) activity are part of an intentionally designed cellular defense mechanism against metal toxicity. This challenges the conventional understanding of the Aβ peptide in the AD pathogenesis. It is not until this BACE1 system, primarily the associated amyloid plaque deposit sites, are saturated with heavy and other metals and the environmental exposure to these cations continues to influx oxidative ions into the brain, do the indications of neurodegeneration begin to become symptomatic.

## Evolution of the hypothesis

Contrary to the current AD model that considers BACE1 activity as aberrant, we suggest that increased BACE1 activity and subsequent Aβ production are neuroprotective responses to heavy and other metal toxicity.

BACE1 activation is directly related to the escalation of NF-kB signaling – the inflammatory signaling pathway (14). As inflammatory signaling escalates BACE1 activity is triggered to escalate as well (13).

It is proposed that this incremental inflammatory activity is reflective of heavy or other metal toxicity and the incremental oxidative activity it promotes. Oxidation and inflammation go hand in hand; one facilitating the other in a cyclical fashion as we have proposed (24, 25).

This hypothesis posits that Aβ peptides chelate and sequester free metals, forming extracellular amyloid plaques. These plaques can be made up of a variety of Aβ peptide types or species reflecting the different peptides generated by differing processes and conditions, including mutations (PSEN) (25).

Additionally, Aβ may cross cell membranes to chelate intracellular metals, subsequently exporting these complexes to the extracellular space for sequestration where the neuron can be protected from the oxidative effects (26).

Furthermore, it is suggested herein that this chelation and sequestration system can work for long periods in one’s life to protect the brain from oxidative stress until the deposit sites- plaques – become too numerous and saturated with heavy or other metals. At this late stage in the disease evolution, the metal load begins to have a more deleterious effect on the brain resulting in intracellular TAU disruption and irreversible neuron damage leading to apoptosis.

The amyloid plaque is a healthy disposal site; while the TAU-specific anomalies or pathology (hyperphosphorylation and aggregation) are indicative of the progressed state of neurodegeneration (27). It is proposed that each of us has individual predispositions for tolerating such oxidative activity characterized by the rate and type (genetically influenced as well) of Aβ peptide processing and the degree of functionality of endogenous antioxidant systems modulated by Nrf2 transcription which is responsible for catalase, glutathione peroxidase, heme-oxygenase and superoxide dismutase generation. Additionally, the level and duration of heavy metal exposure all play a role in producing a variable pathological risk for clinical symptom manifestation from one person to another.

TAU is a microtubule protein that stabilizes the microtubule to serve as a cytoskeleton anchoring organelles (28). The microtubule system also serves as the cell’s ‘railway system’ for the movement or trafficking of materials within the cell (29). In the context of the proposed paradigm, as intolerance for heavy metal load progresses due to the multifactorial condition, oxidative activity can escalate to advance neuroinflammation, TAU protein hyperphosphorylation and subsequent microtubule disruption.

Continuing in the context of the proposed paradigm: At this advanced stage of the AD pathology when the microtubule system is interrupted by TAU hyperphosphorylation, the Aβ protein is no longer translocated due to interruption of trafficking. It now accumulates intracellularly facilitating hyper-oxidative ROS levels and contributes to irreversible neuron apoptosis.

The pathology of Traumatic Brain Injury (TBI) also results in exposure to metal. Hemorrhage in and around the injured brain area results in iron deposition and a progressive neurodegenerative process as a result of the metal exposure (30). TBI is intimately associated with development of Aβ plaques just like those found in Alzheimer’s disease (29, 31). We proposed this as a factor of concern in our 2015 paper with more recent work expressed in the public domain today by Tang et al. demonstrating such. Historical research has shown that cellular distribution of iron in AD brains is also similar to that in TBI (32).

Neuroinflammation and iron aggregation are characteristic conditions of neurodegenerative diseases like AD, Parkinson’s disease and common TBI (33). Ultimately it is shown that abnormal iron homeostasis induces hydroxyl radical production, and the elevated oxidation subsequently results in aberrant structure and function in the brain; and escalated inflammatory activity (34). Iron is detected with significance in post-mortem analysis of AD brains; and herein hemoglobin binds with the Aβ peptide and localizes in the amyloid plaque as expected (35). Iron chelation to Aβ also enhances neurotoxicity (36) of the Aβ peptide likely because of the oxidative activity the complex bears before it is sequestered within the plaque. Amyloid plaques harbor iron, copper and zinc (37, 38).

## Hypothesis testing by way of meta-analysis

Compelling evidence supports this protective role of Aβ. Studies show that as much as a third of older healthy adults show significant Aβ plaque deposition in the brain (39). These deposits precede and are even considered independent of declines in cognitive deficits. Studies definitively show that heavy and other metals interact intimately with Aβ peptide in various ways including in solution and in the cell membrane (40, 41). Heavy metals are implicated in AD pathology and AD-like disease pathologies. Even metals as ubiquitous as copper are shown to chelate the Aβ peptide and when removed from the Aβ peptide cause inhibition of Aβ assembly (42) falling in perfect alignment with our proposed theory that positions Aβ as a component of an intentionally designed protective chelation system.

Our 2015 published theory leads us to dive deeper today into the analysis of the amino acid sequence of the Aβ peptide revealing some unique supportive features. The first half of the Aβ peptide sequence, as seen in Figure 1, is designed for chelation. Independent research diving into the peptide’s first 16 amino acids shows that this section alone of the peptide can bind up to four copper ions (II) (40). The Aβ peptide is derived from the precursor protein, amyloid precursor protein (APP), as a function of two cleavages by two enzymes. The 42 and 43 amino acid Aβ peptide, Aβ42 and Aβ43, are the more amyloidogenic and pathogenic peptides. These two, Aβ42 or Aβ43, polymerize rapidly and are highly associated with AD (43). If we look above in (44) Figure 1 at the BACE1-generated Aβ42, we see a 42 amino acid peptide (and others) each with unique features.

**Figure 2:**
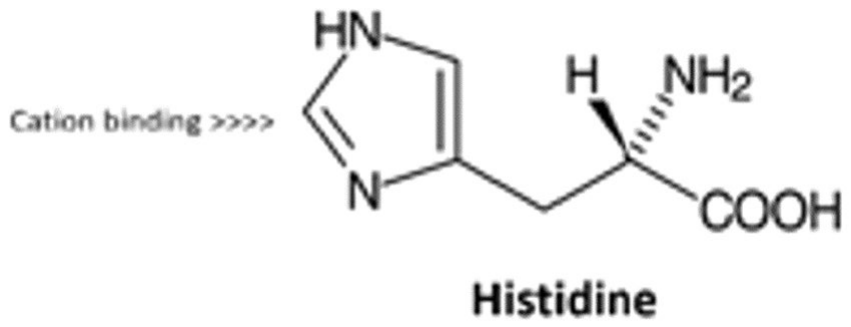
Histidine residue capturing by way of cross-linking, the divalent cation forming a bridge.

They each have a hydrophobic moiety expressed in bold (GAIIGLMVGGVVIA) that facilitates aggregation of peptides in aqueous solution. The tail end on the C-terminal end of the peptide has a long hydrophobic region where G (Glycine) is intermingled as the only hydrophilic inclusion (44, 45). We also have a segment of the peptide that boasts a charged amino acid section (DAEFRHDSGYEVHHQK), which also includes the two side-by-side histidine amino acids previously discussed in Figure 1 and highlighted mechanistically in Figure 2.

In fact, research by Faller and Hureau (45) has demonstrated a probability of copper, iron and even zinc co-ordination with the tail ends of the key amino acids in this hydrophobic segment while these ions have been shown to be intimately involved in the AD pathology (19, 46, 47, 49). This proposed theory showcases this amino acid-metal cross bridging (48) as a protective mechanism.

Showcases the different domains of the BACE1-Generated (*β*-Secretase Enzyme) A*β* peptide associated with metal ion chelation and those with hydrophobic activity known to facilitate aggregation and Beta sheet formation (46). In particular, the side-by-side histidine (13 and 14) residues formed bridges through metal cross-linking providing insight on another feature promoting the aggregation of A*β* protein referred to in the medical community as ‘pathogenic aggregation’ (50), but considered by this paradigm to be an intended biological design to protect the brain from oxidation (including Fenton Reaction).

### Histidine residue capturing by way of cross-linking, the divalent cation forming a bridge

**Figure 3:**
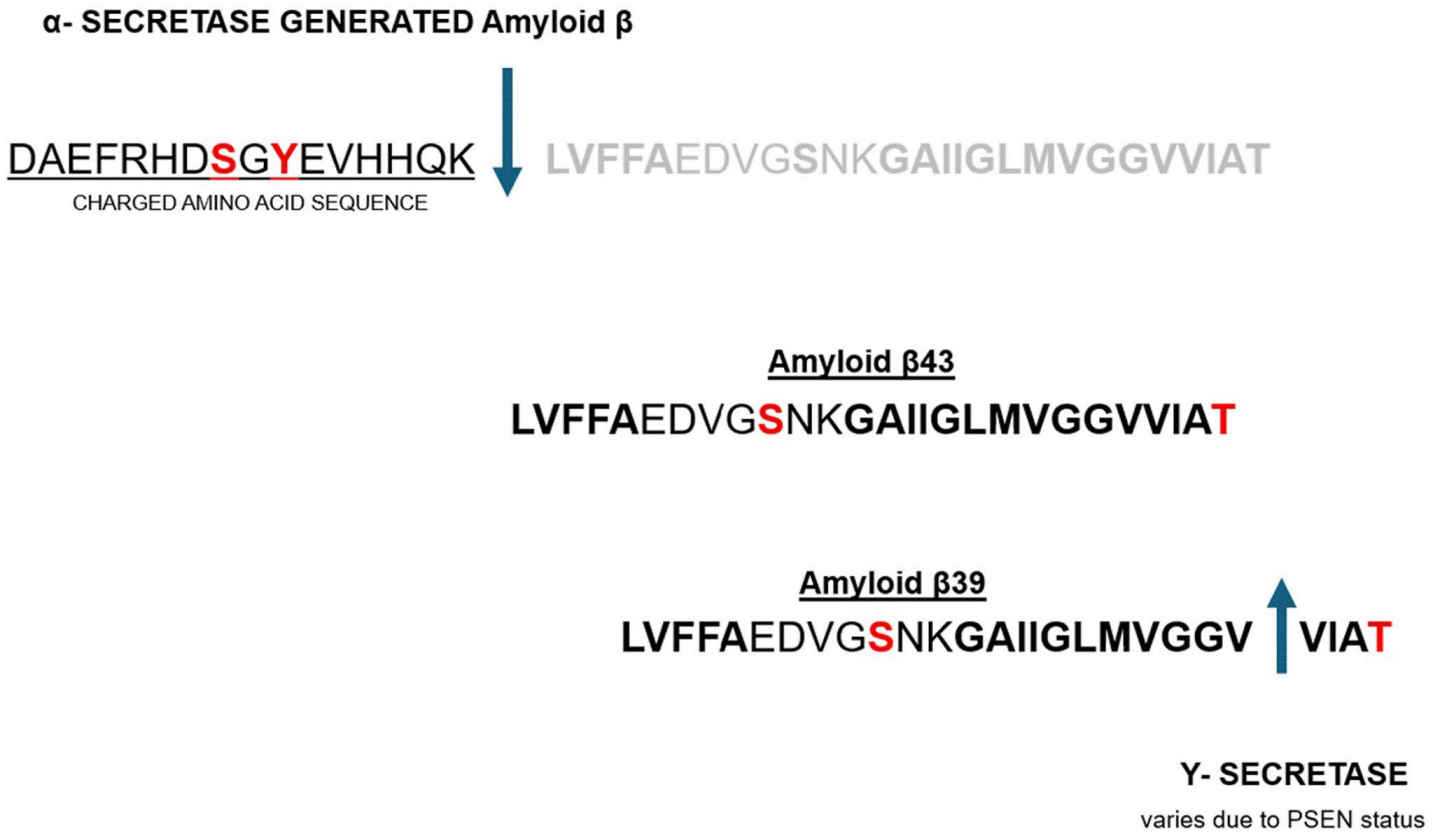
Non-amyloidogenic pathway to A*β* peptide processing leading to omission of the charged amino acid segment with chelation properties (52, 53).

Ultimately, there are two pathways that processing of the APP precursor protein can take to final A*β* peptide end product. One is the previously described BACE1 and the downstream *β*-Secretase cleavage as seen in Figure 1. This pathway results in an A*β* peptide that has irrefutable chelation potential as described above. This path is considered the amyloidogenic pathway and conducive to or associated with AD pathology (51). The other pathway is considered non-amyloidogenic and involves cleavage by *α*-Secretase and is portrayed in Figure 3, below. This latter path yields an A*β* peptide that voids the charged amino acid segment and voids the histidine repeats that are intimately associated with metal chelation as portrayed in Figure 3.

Non-amyloidogenic pathway to Aβ peptide processing leading to omission of the charged amino acid segment with chelation properties (52, 53).

The proposed hypothesis underwriting the new paradigm suggests that BACE1 activation by the inflammatory response results in the downstream *β*-Secretase cleavage to produce the biological countermeasure (amyloidogenic peptide) to the excessive oxidation and inflammation. This amyloidogenic peptide is ultimately produced by intentional biological design in response to metal toxicity and the oxidative and inflammatory result of that uncontrolled oxidation. Alternatively, a peptide is produced in the absence of the oxidative and inflammatory activity where NF-kB Signaling would not be heightened. In this latter case α-Secretase cleavage of the APP precursor peptide would ensue. Typically, a ‘normal brain’ will have small levels of A*β* protein. Both enzymes, *β*-Secretase and α-Secretase compete in this ‘normal’ healthy brain but *β*-Secretase prevails as an APP cleavage enzyme if NF-kB Signaling is robustly active (12). As much as 80 percent of AD brains have elevated *β*-Secretase activity and low *α*-Secretase activity (52). Researchers have concluded that this high *β*-Secretase and low *α*-Secretase finding in the AD brain “may account for the means by which the majority of people develop AD” (54, 57).

However, in the context of our paradigm shift the proposed hypothesis positions this a little differently: The heavy and other metal toxicity is the cause of the high β-Secretase activity and low α-Secretase activity dynamic. The resulting Aβ peptide (with charged amino acids) with chelated metals which aggregate as plaque development is the consequential attempt by the body to protect itself.

Looking beyond the peptide and at the plaque, we see studies indicate that Aβ peptide aggregation and plaque formation precede Alzheimer’s symptoms (53–55), suggesting a long-term protective mechanism against metal-induced oxidative damage. This falls in line with our theory. Research by Rogers et al. showcases their discovery of an Iron-Responsive Element (IRE) built into the APP transcript. Further evidence that APP is, in fact, a metalloprotein and this too supports the current hypothesis that metals modulate APP regulation (56). Ashok et al. demonstrate that environmental pollutants can contribute AD pathology – an increase in Aβ in the rat brain and ensuing cognitive deficits as a function of exposure to environmental As, Cd and Pb (57).

Other enzymes involved in Aβ peptide processing include *Υ*-Secretase. Although we have not assigned much attention to Y-Secretase in this paper, it has garnered lots of attention by researchers historically because it is the enzyme that cleaves the Aβ peptide so it can be released. It shortens the Aβ peptide as seen in Figure 3 into what is said to be shorter, ‘stickier’ peptides (58, 59). Other causes of the AD pathology include various mutations that lead to Familial Alzheimer’s Disease (FAD) characterized by early development of Aβ peptide and plaques (58, 59) and the toxicity from these plaques and migrating Aβ peptides may also be associated with the metals that these peptides are spontaneously chelating.

### Implications for AD progression

The proposed paradigm states that neurons can tolerate metal toxicity in the early stages of exposure due to BACE1-mediated Aβ peptide production. However, as AD progresses and heavy metal exposure persists, uncontrolled metal accumulation and reactive oxygen species (ROS) levels overwhelm this protective mechanism. This leads to increased inflammation, Tau protein hyperphosphorylation, and disruption of microtubule stability, eventually causing neuron apoptosis, tissue loss and an exponential advancement of disease.

## Conclusion

This article presents a paradigm shift in our understanding of Aβ-peptides in AD. Rather than being solely pathogenic, Aβ and BACE1 activity may represent strategic cellular defenses against metal toxicity. These findings highlight the need for a reassessment of therapeutic strategies targeting Aβ production, suggesting that inhibition of BACE1 may inadvertently disrupt protective mechanisms and exacerbate disease progression.

This model indicates that the current AD treatment approach of inhibiting BACE1 may not support resolution of disease progression and may, in fact, result in exacerbation. While typical AD treatment protocols also include the application of anti-inflammatory strategies which may mitigate progression of symptoms for a short period, failure to terminate exposure to the environmental sources of oxidative heavy metals will continue to strain the biological system beyond the anti-inflammatory countermeasure especially in the context of BACE1 inhibition.

### Future directions

It is estimated that 5.5 million North Americans suffer from varying degrees of Alzheimer’s disease (AD) and by the year 2050 it may be one in 85 people globally (100 Million). While we believe that metal toxicity plays a significant role in sporadic Alzheimer’s disease, the current literature speaks to the mere involvement of metal ions. Studies and reviewers have yet to link cellular events including known structural changes such as amyloid plaque development to this metal toxicity the way it was originally proposed by us in 2015 and further elaborated here.

Further research is necessary to validate these findings and explore the therapeutic potential of modulating heavy metal toxicity by chelation therapies and by this treatment modality positively affecting A*β* production and BACE1 activity. Understanding the dual roles of Aβ in neuroprotection and neurodegeneration and the central causal role that metal toxicity may be playing could lead to more nuanced and effective treatments for AD.

Research will need to consider how the administration of exogenous ketone body, β-hydroxybutyrate (3-hydroxybutyrate), might sustain neurons as an ATP substrate alternative to glucose while in the advanced state of AD and the type III diabetic condition. AD and type III diabetes share mechanisms that result in co-manifestation (60). These ketone bodies are ATP substrates and do not depend on insulin signaling form uptake, but they are also anti-inflammatory agents that cross the blood brain barrier to serve the type III diabetic brain in many ways (61).

This ketone body administration might support neurons to the point where apoptosis and loss of brain mass is slowed down; while the metal exposure is identified and eliminated. Alternatively, facilitation of endogenous ketone production via the ketogenic diet or the use of other facilitators of ketogenesis such as caprylate or derivatives thereof now in research. However, researchers will need to investigate how robust diagnostic programs can be applied to identify heavy metal toxicity in the brains of AD patients and develop chelation therapy that can effectively remove heavy metal toxicity without disrupting extracellular amyloid plaque sequestration of these metals before too much irreversible damage is done (62, 63). This preventive and interceptive strategy might be the best way to circumvent the progression of AD to dysfunctional states of disease.

## References

[1] Tuppo EE Arias HR. The role of inflammation in Alzheimer’s disease. Int J Biochem Cell Biol. (2005) 37:289–305. doi: 10.1016/j.biocel.2004.07.009, PMID: 15474976

[2] Kinney JW Geyer MA. Inflammation as a central mechanism in Alzheimer’s disease. Alzheimers Dement. (2018) 4:575–90. doi: 10.1016/j.trci.2018.06.014, PMID: 30406177 PMC6214864

[3] Das B Yan R. Role of BACE1 in Alzheimer’s synaptic function. Transl Neurodegener. (2017) 6:1–8. doi: 10.1186/s40035-017-0092-4 28855981 PMC5575945

[4] Koelsch G. BACE1 function and inhibition: implications of intervention in the amyloid pathway of Alzheimer’s disease pathology. Molecules. (2017) 22:1723. doi: 10.3390/molecules22101723, PMID: 29027981 PMC6151801

[5] Hampel H Lütjohann D. The amyloid-β pathway in Alzheimer’s disease. Mol Psychiatry. (2021) 26:5481–503. doi: 10.1038/s41380-021-01249-0, PMID: 34456336 PMC8758495

[6] Cavaleri F. Review of amyotrophic lateral sclerosis, Parkinson’s and Alzheimer’s diseases helps further define pathology of the novel paradigm for Alzheimer’s with heavy metals as primary disease cause. Med Hypotheses. (2015) 85:779–90. doi: 10.1016/j.mehy.2015.10.009, PMID: 26604027

[7] Cavaleri F. Paradigm shift redefining molecular, metabolic, and structural events in Alzheimer’s disease involves a proposed contribution by transition metals. Med Hypotheses. (2015) 84:460–9. doi: 10.1016/j.mehy.2015.01.044, PMID: 25691377

[8] Erecińska M Silver IA. Tissue oxygen tension and brain sensitivity to hypoxia. Respir Physiol. (2001) 128:263–76. doi: 10.1016/S0034-5687(01)00306-1 11718758

[9] Edmond J Johnson R. Dietary cholesterol and the origin of cholesterol in the brain of developing rats. J Nutr. (1991) 121:1323–30. doi: 10.1093/jn/121.9.1323, PMID: 1880610

[10] Martín MG Koval M. Cholesterol in brain disease: sometimes determinant and frequently implicated. EMBO Rep. (2014) 15:1036–52. doi: 10.15252/embr.201439225, PMID: 25223281 PMC4253844

[11] Richard D Trautwein EA. Polyunsaturated fatty acids as antioxidants. Pharmacol Res. (2008) 57:451–5. doi: 10.1016/j.phrs.2008.05.002 18583147

[12] Bazinet RP Layé S. Polyunsaturated fatty acids and their metabolites in brain function and disease. Nat Rev Neurosci. (2014) 15:771–85. doi: 10.1038/nrn3820 25387473

[13] Liu JJ Yang X. Pathways of polyunsaturated fatty acid utilization: implications for brain function in neuropsychiatric health and disease. Brain Res. (2015) 1597:220–46. doi: 10.1016/j.brainres.2014.11.059, PMID: 25498862 PMC4339314

[14] Millot P Pison C. STAT3 inhibition protects against neuroinflammation and BACE1 upregulation induced by systemic inflammation. Immunol Lett. (2020) 228:129–34. doi: 10.1016/j.imlet.2020.10.004 33096140

[15] Chen CH. *Regulation of human BACE1 gene expression by NF-kappa B signaling*. [Doctoral dissertation]. University of British Columbia, UBC Digital Collections. (2007).

[16] Chen CH Wu SH Lin YJ. Increased NF-κB signalling up-regulates BACE1 expression and its therapeutic potential in Alzheimer's disease. Int J Neuropsychopharmacol. (2012) 15:77–90. doi: 10.1017/S1461145711000149, PMID: 21329555

[17] El Assar M Rodríguez-Mañas L. Oxidative stress and vascular inflammation in aging. Free Radic Biol Med. (2013) 65:380–401. doi: 10.1016/j.freeradbiomed.2013.07.003 23851032

[18] Ramos-González E Pérez-Villalba A. Relationship between inflammation and oxidative stress and its effect on multiple sclerosis. Neurologia. (2021) 36:563–4. doi: 10.1016/j.nrl.2021.04.002, PMID: 38553104

[19] Bolognin S Tosi G Valenti D. Aluminum, copper, iron, and zinc differentially alter amyloid-Aβ1–42 aggregation and toxicity. Int J Biochem Cell Biol. (2011) 43:877–85. doi: 10.1016/j.biocel.2011.02.009, PMID: 21376832

[20] Ferreira PC Lima JL. Aluminum as a risk factor for Alzheimer’s disease. Rev Lat Am Enfermagem. (2008) 16:151–7. doi: 10.1590/S0104-11692008000100023 18392545

[21] Gu H Liu W Zhang Z. Increased β-amyloid deposition in Tg-SWDI transgenic mouse brain following in vivo lead exposure. Toxicol Lett. (2012) 213:211–9. doi: 10.1016/j.toxlet.2012.07.002, PMID: 22796588 PMC3461595

[22] Basha MR Reddy A Ali SS. Lead (Pb) exposure and its effect on APP proteolysis and Aβ aggregation. FASEB J. (2005) 19:2083–4. doi: 10.1096/fj.05-4375fje, PMID: 16230335

[23] Bjørklund G Dadar M. Insights into the potential role of mercury in Alzheimer’s disease. J Mol Neurosci. (2019) 67:511–33. doi: 10.1007/s12031-019-01274-3, PMID: 30877448

[24] Wang L Zhang Y. Current understanding of metal ions in the pathogenesis of Alzheimer’s disease. Transl Neurodegener. (2020) 9:1–13. doi: 10.1186/s40035-020-00198-4 32266063 PMC7119290

[25] Azar J Colom L Smith J Singh P. Mercury and Alzheimer’s disease: a look at the links and evidence. Metab Brain Dis. (2021) 36:361–74. doi: 10.1007/s11011-020-00649-5 33411216

[26] Notarachille G Arciola CR. Heavy metals toxicity: effect of cadmium ions on amyloid beta protein 1–42. Possible implications for Alzheimer’s disease. Biometals. (2014) 27:371–88. doi: 10.1007/s10534-014-9719-6, PMID: 24557150

[27] Chatterjee S. Oxidative stress, inflammation, and disease In: Chatterjee S, editor. Oxidative stress and biomaterials. Amsterdam, Netherlands: Elsevier (2016). 35–58.

[28] Kabir MT Zhang M Zhang X. Exploring the role of PSEN mutations in the pathogenesis of Alzheimer’s disease. Neurotox Res. (2020) 38:833–49. doi: 10.1007/s12640-020-00232-x, PMID: 32556937

[29] Jiménez JS. Macromolecular structures and proteins interacting with the microtubule-associated tau protein. Neuroscience. (2023) 518:70–82. doi: 10.1016/j.neuroscience.2022.05.023 35609757

[30] Colnaghi L Meli E Cirulli T. Tau and DNA damage in neurodegeneration. Brain Sci. (2020) 10:946. doi: 10.3390/brainsci10120946, PMID: 33297375 PMC7762255

[31] Chaudhary AR Hendricks AG. Tau directs transport along microtubules through differential regulation of kinesin and dynein. Biophys J. (2017) 112:261a–2a. doi: 10.1016/j.bpj.2016.11.1422

[32] Daglas M Adlard PA. The involvement of iron in traumatic brain injury and neurodegenerative disease. Front Neurosci. (2018) 12:981. doi: 10.3389/fnins.2018.00981, PMID: 30618597 PMC6306469

[33] Smith DH Johnson V. Amyloid β accumulation in axons after traumatic brain injury in humans. J Neurosurg. (2003) 98:1072–7. doi: 10.3171/jns.2003.98.5.1072 12744368

[34] Tang S Chen M. The role of iron, its metabolism, and ferroptosis in traumatic brain injury. Front Cell Neurosci. (2020) 14:590789. doi: 10.3389/fncel.2020.590789, PMID: 33100976 PMC7545318

[35] DeKosky ST Blennow K. Association of increased cortical soluble Aβ42 levels with diffuse plaques after severe brain injury in humans. Arch Neurol. (2007) 64:541–4. doi: 10.1001/archneur.64.4.541, PMID: 17420316

[36] Connor JR Menzies SL. Cellular management of iron in the brain. J Neurol Sci. (1995) 134:33–44. doi: 10.1016/0022-510X(95)00206-H 8847543

[37] Urrutia PJ Castro JP. Inflaming the brain with Iron. Antioxidants. (2021) 10:61. doi: 10.3390/antiox10010061, PMID: 33419006 PMC7825317

[38] Ward RJ Zucca FA. The role of iron in brain ageing and neurodegenerative disorders. Lancet Neurol. (2014) 13:1045–60. doi: 10.1016/S1474-4422(14)70117-6, PMID: 25231526 PMC5672917

[39] Chuang JY Huang CY Chiu PY. Interactions between amyloid-β and hemoglobin: implications for amyloid plaque formation in Alzheimer’s disease. PLoS One. (2012) 7:e33120. doi: 10.1371/journal.pone.0033120, PMID: 22412990 PMC3295782

[40] Wang L Du Y. Iron enhances the neurotoxicity of amyloid β. Transl Stroke Res. (2012) 3:107–13. doi: 10.1007/s12975-011-0099-8 22822413 PMC3401050

[41] Rogers JT Sinha S. Iron and the translation of the amyloid precursor protein (APP) and ferritin mRNAs: Riboregulation against neural oxidative damage in Alzheimer’s disease. Biochem Soc Trans. (2008) 36:1282–7. doi: 10.1042/BST0361282, PMID: 19021541 PMC2746665

[42] Uranga RM Salvador GA. Unraveling the burden of iron in neurodegeneration: intersections with amyloid beta peptide pathology. Oxidative Med Cell Longev. (2018) 2018:2850341. doi: 10.1155/2018/2850341, PMID: 29581821 PMC5831758

[43] Rodrigue KM Kennedy K. Beta-amyloid deposition and the aging brain. Neuropsychol Rev. (2009) 19:436–50. doi: 10.1007/s11065-009-9118-x, PMID: 19908146 PMC2844114

[44] Tõugu V Säälik K. Interactions of Zn (II) and cu (II) ions with Alzheimer’s amyloid-beta peptide: metal ion binding, contribution to fibrillization, and toxicity. Metallomics. (2011) 3:250–61. doi: 10.1039/c0mt00073f, PMID: 21359283

[45] Faller P Hureau C. Role of metal ions in the self-assembly of the Alzheimer’s amyloid-β peptide. Inorg Chem. (2013) 52:12193–206. doi: 10.1021/ic4003059 23607830

[46] Faller P. Copper and zinc binding to amyloid-β: coordination, dynamics, aggregation, reactivity, and metal-ion transfer. Chembiochem. (2009) 10:2837–45. doi: 10.1002/cbic.200900321, PMID: 19877000

[47] Geng J Yang S Xu G. Liberation of copper from amyloid plaques: making a risk factor useful for Alzheimer’s disease treatment. J Med Chem. (2012) 55:9146–55. doi: 10.1021/jm3003813 22663067

[48] Welander H Nilsson LN. Aβ43 is more frequent than Aβ40 in amyloid plaque cores from Alzheimer disease brains. J Neurochem. (2009) 110:697–706. doi: 10.1111/j.1471-4159.2009.06170.x, PMID: 19457079

[49] Bag S Singh M Gupta R. Hydrophobic tail length plays a pivotal role in amyloid beta (25–35) fibril–surfactant interactions. Proteins. (2016) 84:1213–23. doi: 10.1002/prot.25069 27192507

[50] Condron M Thiyagarajan M Bitan G. Synthesis and purification of highly hydrophobic peptides derived from the C-terminus of amyloid β-protein. Open Biotechnol J. (2008) 2:87–93. doi: 10.2174/1874070700802010087, PMID: 19898686 PMC2773559

[51] Faller P Hureau C. Bioinorganic chemistry of copper and zinc ions coordinated to amyloid-β peptide. Dalton Trans. (2009) 7:1080–94. doi: 10.1039/B900969G 19322475

[52] Sinha S Anderson JP Barbour R Basi GS Caccavello R Davis D. Purification and cloning of amyloid precursor protein β-secretase from human brain. Nature. (1999) 402:537–40. doi: 10.1038/990114, PMID: 10591214

[53] Hampel H Reymann J. The β-secretase BACE1 in Alzheimer’s disease. Biol Psychiatry. (2021) 89:745–56. doi: 10.1016/j.biopsych.2020.02.001, PMID: 32223911 PMC7533042

[54] Skovronsky DM Lee VM-Y. Protein kinase C-dependent α-secretase competes with β-secretase for cleavage of amyloid-β precursor protein in the trans-Golgi network. J Biol Chem. (2000) 275:2568–75. doi: 10.1074/jbc.275.4.2568, PMID: 10644715

[55] Tyler SJ Wang H. α-And β-secretase: profound changes in Alzheimer’s disease. Biochem Biophys Res Commun. (2002) 299:373–6. doi: 10.1016/S0006-291X(02)02635-9 12445809

[56] Rogers JT Randall JD Cahill CM Eder PS Huang X Gunshin H. An Iron-responsive element type II in the 5′-untranslated region of the Alzheimer’s amyloid precursor protein transcript. J Biol Chem. 277:45518–28. doi: 10.2174/1389450043345272 12198135

[57] Ashok A Rai NK Tripathi S Bandyopadhyay S. Exposure to as, cd, and Pb-mixture induces Aβ, amyloidogenic APP processing and cognitive impairments via oxidative stress-dependent neuroinflammation in young rats. Toxicol Sci. (2015) 143:64–80. doi: 10.1093/toxsci/kfu208, PMID: 25288670

[58] Baranello J Liu M. Amyloid-beta protein clearance and degradation (ABCD) pathways and their role in Alzheimer’s disease. Curr Alzheimer Res. (2015) 12:32–46. doi: 10.2174/1567205012666141218140953, PMID: 25523424 PMC4820400

[59] Hur JY. γ-Secretase in Alzheimer’s disease. Exp Mol Med. (2022) 54:433–46. doi: 10.1038/s12276-022-00754-8 35396575 PMC9076685

[60] Kandimalla R Thirumala V Reddy PH. Is Alzheimer’s disease a type 3 diabetes? A critical appraisal. Biochim Biophys Acta Mol Basis Dis. 1863:1078–89. doi: 10.1016/j.bbadis.2016.08.018 PMC5344773 27567931

[61] Moller N. Ketone body, 3-Hydroxybutyrate: minor metabolite - major medical manifestations. J Clin Endocrinol Metabol. (2020) 105:2884–92. doi: 10.1210/clinem/dgaa370, PMID: 32525972

[62] Han D Li X Wang T. Molecular modeling of zinc and copper binding with Alzheimer’s amyloid β-peptide. Biometals. (2008) 21:189–96. doi: 10.1007/s10534-007-9107-6, PMID: 17629774

[63] Cole SL Vassar R. The role of amyloid precursor protein processing by BACE1, the β-secretase, in Alzheimer disease pathophysiology. J Biol Chem. (2008) 283:22885–9. doi: 10.1074/jbc.R800023200, PMID: 18650431 PMC2662048

